# Post-COVID-19 Acute Transverse Myelitis: A Case Report and Literature Review

**DOI:** 10.7759/cureus.20628

**Published:** 2021-12-22

**Authors:** Rija Qazi, Ayesha Memon, Alaa S Mohamed, Muneeba Ali, Romil Singh

**Affiliations:** 1 Neurology, Ziauddin College of Medicine, Karachi, PAK; 2 Neurology, Augusta University, Augusta, USA; 3 Internal Medicine, Foundation University Medical College, Islamabad, PAK; 4 Critical Care, Mayo Clinic, Rochester, USA

**Keywords:** longitudinally extensive transverse myelitis, myelopathy, acute transverse myelitis (atm), covid 19, sars-cov-2

## Abstract

Acute transverse myelitis (ATM) following coronavirus disease 19 (COVID-19) is rarely reported in the literature. We report a case of ATM after COVID-19 infection in a female who presented with sudden onset bilateral lower limb weakness, paresthesia, and urinary retention. She developed fever, cough, dyspnea two weeks ago, and her COVID-19 test was positive one week later. After a complete physical examination and detailed investigations, including cerebrospinal fluid analysis, autoimmune screening, and infectious workup, a diagnosis of ATM due to COVID-19 was made. Magnetic resonance imaging of the whole spine confirmed the diagnosis of ATM. She was managed with intravenous methylprednisolone, physical therapy, and bladder training and her condition improved gradually.

## Introduction

Coronavirus disease 19 (COVID-19) primarily affects the lungs, and a growing body of evidence suggests that COVID-19 may have multisystem involvement during acute and post-infection [1]. The severe acute respiratory distress syndrome 2 (SARS-CoV-2), the causative agent of COVID-19, also affects the nervous system and can present with a wide range of clinical manifestations and complications, which can rapidly progress and need immediate evaluation and intervention [2-5]. However, acute transverse myelitis (ATM) following COVID-19 is not widely reported [6-11]. Herein we describe a case of an ATM after COVID-19 infection.

## Case presentation

A 35-year-old female with a past medical history of hypothyroidism was brought to the emergency department with sudden onset bilateral lower limb weakness. She reported that she could not get up from the bed in the morning, followed by urinary retention and abnormal sensations in the lower part of the body up to the thorax level. Two weeks ago, she developed a high-grade fever, dyspnea, cough, and loss of smell, and her symptoms persisted. Her nasopharyngeal swab for COVID-19 was positive one week later, and she was commenced on azithromycin. Her symptoms resolved gradually, and she developed neurological symptoms today. She was diagnosed with hypothyroidism five years back and compliant with her medications. She had no history of trauma, joint pain, alcohol abuse, or illicit drug use. 

On examination, she was afebrile with normal vitals. She looked anxious and well oriented in time, place, and person. On auscultation, she had bilateral rhonchi, and cardiovascular examination was unremarkable. Suprapubic tenderness was noted on abdominal examination. Neurological examination revealed paresthesia and hypoesthesia bilaterally below the nipple. Cranial nerves were intact, and there were no meningeal signs. Hypertonia and hyperreflexia were noted in both lower limbs, and she had the power of 2/5 and 5/5 in lower limbs and upper limbs, respectively. These findings were suggestive of upper motor neuron lesions. A foley catheter was inserted, and one liter of urine was drained.

The results of initial investigations were nonsignificant except for elevated c-reactive protein and d-dimer (Table 1). 

**Table 1:** Result of initial investigations WBC: white blood cell, BUN: blood urea nitrogen, ESR: erythrocyte sedimentation rate, CRP: c-reactive protein, Hb: Hemoglobin, RBC: red blood cell.

Parameter	Lab value	Reference range
WBC	8000 cells/mm^3^	4000-10,000
RBC	4.2 million cells/mm^3^	4.1-5.3
Platelet count	191,000 cells/mm^3^	150,000-350,000
Hb	12.1 mg/dl	12-15
BUN	19 mg/dl	8-20
Creatinine	1.1 mg/dl	0.7-1.2
ESR	29	
CRP	29 mg/L	< 05
D-dimer	0.9 mg/L	< 0.5
Blood glucose	189 mg/dl	< 200

**Figure 1:**
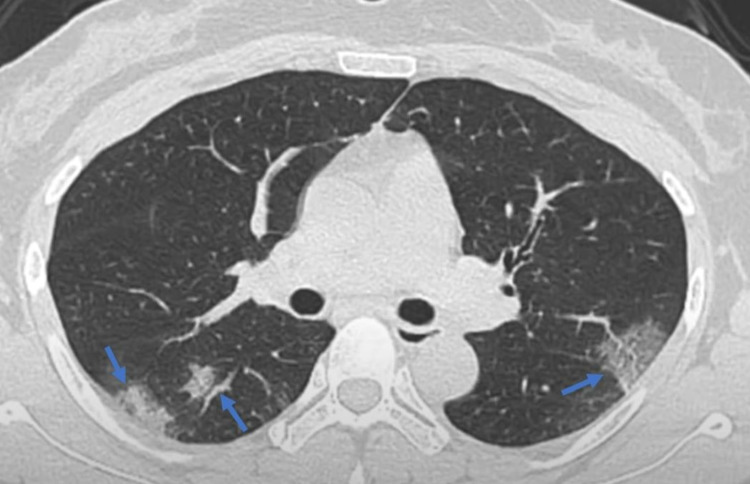
CT chest showing patchy opacities in both lungs

**Figure 2:**
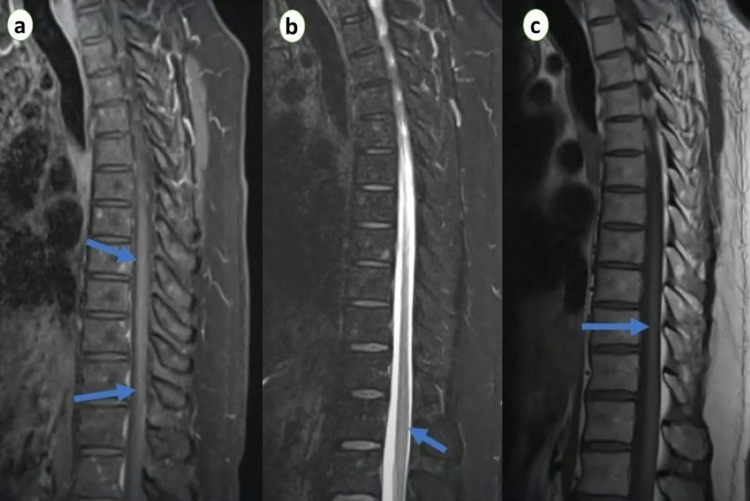
MRI thoracic spine: (a) Sagittal T1 sequence with gadolinium contrast. (b) Sagittal T1 sequence of the thoracic spine. (c) Sagittal T2 sequence showing a high signal intensity starting from the T2 level.

High-resolution chest computed tomography (CT) revealed peripheral areas of patchy opacities and consolidation in both lungs (Figure 1). Magnetic resonance imaging (MRI) of the whole spine revealed a long nodular segment of T2-weighted signal elevation centrally in the spinal cord starting from T-2 level without the presence of mass or significant enhancement (Figure 2). MRI brain was normal and did not show any recent or ongoing inflammatory changes. The results of the cerebrospinal fluid analysis did not show any significant abnormality (Table 2). Autoimmune serology for antinuclear antibodies, rheumatoid factor, and other atypical antibodies, including anti-APQ-4 antibodies, were negative. Infectious and viral workups, including human immunodeficiency virus, varicella-zoster, herpesvirus, cytomegalovirus, and syphilis were also negative except for COVID-19.

**Table 2:** Result of CSF analysis

Spinal fluid	Result	Reference range
Opening pressure	14	05-20 cmH_2_O
White blood cell	2	<5 mm^3^
Red blood cell	1	<5 mm^3^
Protein	40	15-45 mg/dl
Glucose	47	40-75 mg/dl
Gram stain	Negative	Negative
Lactate dehydrogenase	08	1/10
Albumin	61	56-79%
Color	Colorless	Colorless
Appearance	Clear	Clear

Based on the history and detailed investigations, acute myelitis was diagnosed as a direct injury to the spinal cord or the sequelae of the post-infectious process of COVID-19. She was commenced on intravenous methylprednisolone 1g/day for seven days and physiotherapy. Bladder training was also initiated. She reported resolution of symptoms and was discharged with follow-up. On a recent follow-up, she continued to improve, and her recent spine MRI showed resolution of the changes seen previously. 

## Discussion

COVID-19 primarily affects the respiratory system, and involvement of the nervous system has also been reported. Neurological manifestations and complications include headaches, seizures, encephalitis, stroke, dizziness, ataxia, and neuropathies [12]. ATM is a rare complication of COVID-19 and characterized by inflammation of the spinal cord leading to spinal cord dysfunction below the lesion level. We have tabulated the cases of ATM following COVID-19 infection in the USA (Table 3). The cervical region is most involved, followed by the thoracic region [13]. ATM can be compressive and non-compressive myelopathies, and non-compressive ATM is caused by autoimmune, vaccination, infection, and radiation [14]. The onset of ATM may be acute within hours to a day and subacute from days to weeks depending upon the etiology.

**Table 3:** Cases with ATM following COVID-19 infection F: female, T: thoracic, LEATM: longitudinally extensive acute transverse myelitis, ADEM: acute disseminated encephalomyelitis, M: male, C: cervical.

Author	Sex/age (year)	COVID-19 Clinical manifestations	Latency period (days)	Lesion level	Clinical features	Management
Valiuddin et al. [6]	F/61	Afebrile, rhinorrhea, chills	7	LEATM, C1-T1	Quadriparesis, hyporeflexia, incontinence	methylprednisolone
Durrani et al. [7]	M/24	Fever, pneumonia	14	LEATM, T7-12	Paraplegia, incontinence	methylprednisolone
Utukuri et al. [8]	M/44	Paraparesis, leg numbness	2	LEATM, C5-T6	Urinary retention, ataxia	methylprednisolone
McCuddy et al. [9]	F/40	Fever, pneumonia	7	ADEM	Paraplegia, hyperreflexia	methylprednisolone
Kaur et al. [10]	F/3	asymptomatic	21	LEATM, C1-T6	Areflexia, incontinence	methylprednisolone
Kara et al. [11]	F/39	Fever, flu-like symptoms	21	C5-T12	Paresthesia, hyperreflexia	methylprednisolone

Pathophysiology of ATM due to COVID-19 is due to neurotropic properties of SARS-CoV-2. The novel virus enters the nervous system through the nasopharyngeal area by using angiotensin-converting enzyme receptor 2 (ACE2) receptors present in the nervous system, including glial cells and basal ganglia [15]. A possible mechanism for post-infectious ATM is molecular mimicry, where immune-mediated injury to the nervous system occurs due to the production of autoantibodies [10,16]. In addition, a severe inflammatory response by inflammatory mediators and cytokine and microangiopathy induced by cytokine and complement activation can also lead to ATM.

ATM is challenging to diagnose, and it is often the diagnosis of exclusion, primarily when it is associated with non-compressive myelopathies. MRI is the imaging modality of choice and is not limited to only cord lesions but may also exclude other pathologies associated with cord lesions [12]. Affected segments of the spinal cord appear hyperintense on T2-weighted images and show variable contrast enhancement and swelling. Management of ATM depends on etiology, duration, and severity of symptoms and is treated with steroids, antivirals, or immunoglobulins [17]. Patients with early diagnosis and immediate management usually show a favorable prognosis. This article described a rare case of ATM following COVID-19 infection treated early with methylprednisolone. 

## Conclusions

COVID-19 with its neurotropism could trigger molecular mimicry and induce various neurological complications. Our study highlights a case of ATM, a rare complication of COVID-19. ATM should be considered as a differential diagnosis for patients with SARS-CoV-2 infection who present with a neurological deficit. It is critical to establish a diagnosis and treatment strategy early to avoid serious complications. Additionally, our case needs further study to establish the causality, and more studies are required to verify the association between COVID-19 infection and ATM.
